# Role of interferon regulatory factor-mediated signaling in psoriasis

**DOI:** 10.7150/ijms.61973

**Published:** 2021-10-16

**Authors:** Wen-Ming Wang, Feng Li, Hong-Zhong Jin

**Affiliations:** Department of Dermatology, State Key Laboratory of Complex Severe and Rare Diseases, Peking Union Medical College Hospital, Chinese Academy of Medical Science and Peking Union Medical College, Beijing, China.

**Keywords:** Psoriasis, Type I Interferons, Toll-Like Receptors, Interferon Regulatory Factors

## Abstract

Psoriasis is a chronic inflammatory disease that involves both the innate and adaptive immune systems. Type I interferons (IFNs), the production of which is partially regulated by toll-like receptors (TLRs), play an important role in the pathogenesis of psoriasis, especially psoriasis caused by skin trauma, known as the Koebner phenomenon. IFN regulatory factors (IRFs) function in both innate and adaptive immune responses, and their effect is associated with the regulation of type I IFNs. In this review, we focus on recent advances in understanding the expression of TLRs, IRFs, and type I IFNs in psoriasis. We also highlight the interplay among TLRs, IRFs, and type I IFNs.

## Introduction

Psoriasis is a chronic inflammatory disease that commonly manifests as erythematous plaques with adherent silvery scales 1. The causes of psoriasis are complex, and they include psoriasis autoantigens, the innate and adaptive immune systems, psoriasis-associated susceptibility genes, and environmental factors 1, 2. Type I interferons (IFNs; IFN-α and IFN-β), in addition to acting as crucial antiviral factors, have also been shown to function in the pathogenesis of psoriasis 3. The family of IFN regulatory factor (IRF) transcription factors is involved in regulating IFN gene expression 4. In mammals, nine IRFs have been identified (IRF1-9), all of which play important roles in both innate and adaptive immune responses 5. Furthermore, IRFs have been found to be associated with the activation and differentiation of immune cells 5, 6. In this review, we discuss the interplay among IRFs, type I IFNs, and immune cells in psoriasis and the potential value of targeting these factors for future psoriasis treatments.

## Toll-like receptors (TLRs), IRFs, and type I IFNs

Pattern recognition receptors (PRRs) are crucial for innate immunity, and they help activate the adaptive immune system. PRRs can trigger signaling cascades in response to two sources of stimulation: pathogen-associated molecular patterns (PAMPs) from microbes and endogenous danger-associated molecular patterns (DAMPs) released from necrotic cells. The most notable PRRs are toll-like receptors (TLRs), which play an important role in the production of proinflammatory cytokines, including type I IFNs 7-9.

In humans, there are 11 TLR subtypes, TLR1-11. TLR1, TLR2, TLR4, TLR5, TLR6, and TLR10 are extracellular receptors generally localized at the cell plasma membrane, whereas TLR3, TLR7, TLR8, and TLR9 are localized in the endosomal compartments 10, 11. The ligands for TLR10 and TLR11 have not yet been defined 12. TLRs can recognize a wide range of cell surface components derived from bacteria, viruses, fungi, and parasites; for example, TLR1, TLR2, and TLR6 recognize lipoprotein, TLR4 recognizes lipopolysaccharide, TLR5 recognizes flagellin, TLR3 recognizes double-stranded (ds) RNA, TLR7 and TLR8 recognize single-stranded (ss) RNA, and TLR9 recognizes CpG containing unmethylated DNA 11, 13-15.

There are two ways that TLRs can activate adaptive immune responses: through the adaptor MyD88 pathway or through the TRIF pathway. All the TLRs, except for TLR3, signal through the MyD88 pathway. TLR4 can operate through both the TRIF and MyD88 pathways 11. Previous studies have found that TLRs can trigger the induction of type I IFN and cytokine genes through both the TRIF and MyD88 pathways after recognizing a variety of endogenous DAMPs released from necrotic cells and PAMPs 16, 17. As an adaptor protein, MyD88 can lead to gene expression via IRF-1, IRF-5, and IRF-7, whereas TRIF, together with nuclear factor kappa-light-chain-enhancer, can lead to the activation of IRF-3 18-21. While IRF3 is expressed by a variety of cell types, IRF7 is expressed mainly in plasmacytoid DCs (pDCs). The expression of IRF7 can also be induced by type I IFNs; there is a positive feedback effect between IRF7 and type I IFNs 22-24. TLR3, TLR4, TLR7, and TLR9 can induce the expression of both IRF3 and IRF7 23-25. Additionally, previous studies have revealed that IRF3, IRF5, IRF7, and IRF8 can induce the expression of type I IFN genes 23.

## TLR signaling and psoriasis

A study conducted on eight samples of normal skin and 15 samples of lesional and non-lesional biopsies from patients with psoriasis demonstrated that TLR1, TLR2, and TLR5 are constitutively expressed in the keratinocytes of normal skin. It also found that cytoplasmic TLR1 is highly expressed on basal keratinocytes in normal skin, whereas nuclear TLR1 is found in the upper layers of both non-lesional and lesional psoriatic keratinocytes. Furthermore, cytoplasmic TLR2 was mainly detected on basal keratinocytes in normal skin but was found to be highly expressed by the upper layer of epidermis in psoriasis lesions. In general, TLR1 and TLR2 were expressed in the upper and middle epidermis of psoriasis lesions. Notably, the expression levels of TLR1 and TLR2 were significantly lower after adalimumab treatment 26.

The concentration of soluble (s)TLR2 in psoriasis patients, both before and after Goeckerman therapy, was lower than that in healthy controls, whereas the membrane levels of TLR2 on monocytes and granulocytes were significantly upregulated in patients with psoriasis both before and after Goeckerman therapy compared with healthy controls 27. Consistent with this finding, another study also determined that the TLR2 and TLR4 levels were higher in patients with psoriasis than in control subjects 28. Furthermore, immunohistochemical staining revealed that TLR4 expression was upregulated in samples from patients with guttate psoriasis compared with samples from individuals with plaque psoriasis or normal skin 29. The level of TLR7 mRNA in lesional skin from patients with psoriasis was significantly higher than that in healthy controls 30. Real-time polymerase chain reaction (PCR) revealed that the expression levels of TLR3, TLR5, TLR6, TLR7, TLR9, and TLR10 in lesional skin were higher than those in peripheral blood mononuclear cells (PBMCs) of the same patients with psoriasis 31. The study also clarified that the levels of TLR1, TLR8, and TLR10 mRNA in PBMCs from patients with psoriasis were significantly higher than those in PBMCs from healthy controls 31. The expression level of TLR9 in psoriatic lesional skin was higher than that in psoriatic non-lesional skin 32. In addition, TLR9 also can be induced by wounding in lesional psoriatic skin 33. The expression level of TLR5 was lower in basal keratinocytes within a psoriasis lesion compared with that in non-lesional psoriatic keratinocytes 34. Consistent with this, the TLR5 mRNA level was lower in patients with psoriasis than in healthy controls 31. In conclusion, elevated expression levels of TLR1, TLR2, TLR4, TLR7, and TLR9 may facilitate the occurrence of psoriasis.

A study conducted on 175 patients with psoriasis and 170 healthy controls found that genotype and allele frequencies for Arg753Gln TLR2 and -1237 T/C TLR9 gene polymorphisms in patients with psoriasis were similar to those in healthy controls. However, the patients with late onset psoriasis were more likely to carry allele G in the Arg753Gln TLR2 polymorphism, whereas allele T in the -1237 T/C TLR9 polymorphism was statistically more frequent in the patients with early onset psoriasis 35. Another study revealed that the TLR2-rs4696480 AA genotype is related to a higher risk of developing psoriasis in the Turkish population 36. In contrast, the TLR2-rs3804099 single nucleotide polymorphism (SNP) was associated with a higher risk of developing psoriasis vulgaris in the Chinese population 37.

## IRFs and psoriasis

IRF1 is one of the transcription factors regulating type I IFN responses. Although SNPs of the IRF1 promoter may be related to the T-helper type 1 (Th1) response in chronic hepatitis C infection 38, studies have found no association between SNPs of the IRF1 promoter and the pathogenesis of psoriasis 39. IRF1 has been observed throughout the epidermis in both normal skin and psoriatic lesions 40, but one study reported that IRF1 expression is lower in psoriatic lesions than in non-lesions of patients with psoriasis or normal skin. Furthermore, that study also found that the ability of IRF1 and STAT‐1α to activate psoriatic keratinocytes was lower than that of normal keratinocytes after IFN-γ stimulation 41. In contrast, a different study showed that IRF1 was not differentially expressed between psoriatic lesions and healthy skin 42.

IRF2 can suppress the transcription of type I IFN-inducible genes, and it can also inhibit the function of IRF1 under certain circumstances 43. Previous studies have classified psoriasis into two subgroups: type 1 and type 2 44, 45. Type 1 psoriasis refers to cases in which the patient has a low age at onset (early onset) and a positive family history. IRF-2 is considered a potential susceptibility gene for psoriasis, especially type 1 psoriasis 46, 47. IRF-2^+/-^ mice (a haploinsufficiency model) showed more severe psoriasis-like dermatitis compared with wildtype mice 48. IRF2 knockout mice show hyper-responsiveness to type I IFN signaling and exhibit lesions resembling human psoriasis 49. IRF2 is expressed in the basal cell layer of normal skin and in both the basal and suprabasal cell layers of psoriatic lesions 40. In contrast, the expression and distribution of IRF2 were found not to differ between individuals with psoriasis and healthy controls 47.

T-helper (Th)17 cells play an important role in the pathogenesis of psoriasis 50. Previous research demonstrated that T-helper cells from IRF4-deficient mice failed to differentiate into Th17 cells and suggested that IRF4 might play a crucial role in T-helper cell development 51. IRF5 was demonstrated to be essential for supporting Th1 responses after TLR8 stimulation. Furthermore, the cooperation of IRF5 and NF-κB is important for supporting Th17 responses 52. However, although IRF5 is associated with a high risk of developing systemic lupus erythematosus, no relationship between IRF5 polymorphisms and psoriasis per se has been reported 53. In addition, the expression of IRF7 mRNA in psoriatic lesions is significantly higher compared with that in normal skin 54. Consistent with this, RNA-seq also confirmed that IRF7 is significantly upregulated in psoriatic lesions compared with normal skin 42. These findings indicate that IRF might be involved in the pathogenesis of psoriasis.

## Type I IFN signature and psoriasis

The IFN expression triggered in response to viral infection has been studied extensively. IFNs can be classified into three types: type I IFNs (IFN-α and IFN-β), type II IFN (IFN-γ), and type III IFN (IFN-λ) 55. Type I IFNs and type III IFN can be secreted by several cell types 55, whereas type II IFN is released by activated T cells and NK cells 56. Previous studies have indicated that IFNs might play important roles in systemic lupus erythematosus, rheumatoid arthritis, and systemic sclerosis 55, 57, 58.

The “Koebner phenomenon” was first described in psoriasis. This condition is not restricted to psoriasis; however, other skin diseases, such as vitiligo, lichen planus, and verruca vulgaris, can also be associated with the Koebner phenomenon. It is still unknown why a cutaneous trauma (e.g., wound, burn, surgical incision, and tattoo) is able to trigger the pathogenesis of psoriasis in healthy skin 59. Studies have shown that after skin trauma, pDCs can rapidly infiltrate the skin, and IFN-α derived from pDCs may be both necessary and sufficient to trigger psoriasis 60. In a xenograft murine model, the elevated IFN-α level occurred prior to the development of psoriatic changes 60, 61.

Keratinocytes are the main source of IFN-β in psoriasis and skin wounds. The IFN-β produced by these cells promotes the activation and maturation of pDCs 59, 62. Higher levels of type I IFN signaling pathway activity were detected in lesional skin compared with non-lesional skin. Additionally, higher mRNA levels of type I IFNs, IFNAR1, and IFNAR2 were also seen in lesional skin than in non-lesional or normal skin 63. Furthermore, higher levels of IFN-α protein can be detected in patients with active psoriasis than in patients with stationary psoriasis or healthy individuals 63, 64. Anti-tumor necrosis factor-α (TNF-α) agents have been used in the treatment of psoriasis for several years. However, there are reports of new-onset or exacerbation of psoriasis occurring after anti-TNF-α therapy; this condition is known as paradoxical psoriasis 65. Conrad et al. reported that anti-TNF treatment can inhibit pDC maturation, thus leading to the overexpression of type I IFN 66. These findings indicate that the innate immune system driven by pDC-derived type I IFN may play a crucial role in paradoxical psoriasis. IFN-α therapy for hepatitis C can also induce an exacerbation of preexisting psoriasis or the development of new-onset psoriasis, but this can be resolved by the discontinuation of therapy 61, 67, 68. Psoriatic lesions can also be induced by the application of topical imiquimod and may be related to the overexpression of IFN-α 69. Together, these data support a crucial role for type I IFNs in triggering psoriasis.

## Conclusion

As the largest organ in the human body and the first line of defense against pathogens and other threats, the skin is constantly exposed to pathogenic or danger factors from the environment. TLRs can initiate innate immune responses following their recognition of PAMPs or DAMPs. Skin injury or infection can induce the expression of TLRs or stimulate TLRs. TLRs can induce the expression of IRFs through MyD88 pathway or through the TRIF pathway. Furthermore, IRFs can promote the expression of type I IFNs. These studies demonstrated that activation of the TLR-IRF-type I IFN signaling pathway plays an important role in the pathogenesis of psoriasis, especially in the triggering and early phases of this disease. However, understanding of the role of the TLR-IRF-type I IFN signaling pathways in psoriasis is still limited. Thus, further studies are needed to evaluate the exact role and uncover the therapeutic potential of targeting the TLR-IRF-type I IFN signaling pathways in psoriasis.

## Figures and Tables

**Figure 1:**
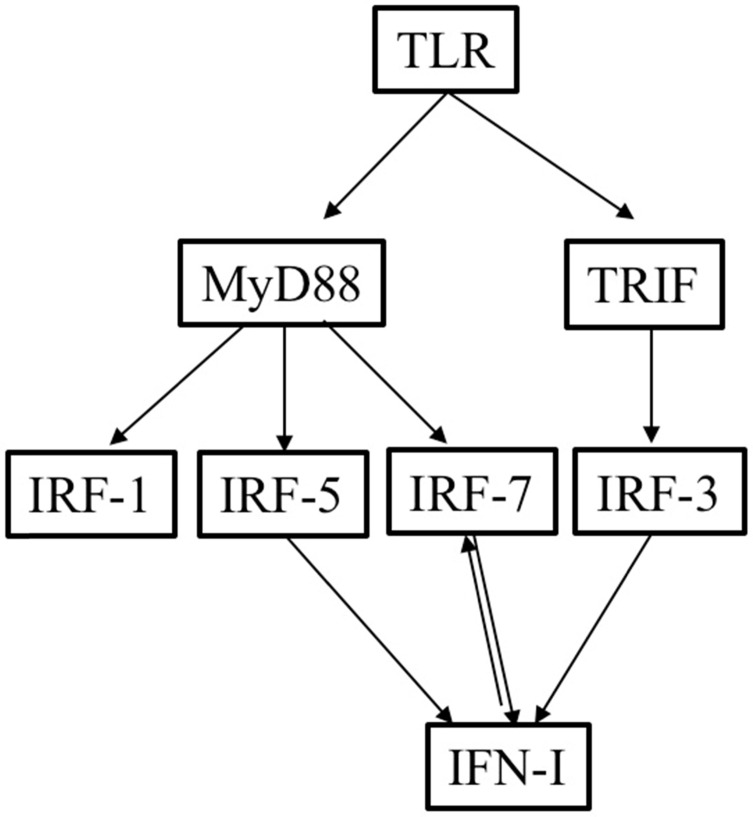
Overview of the relationships among TLRs, IRFs, and type I IFNs.

**Figure 2:**
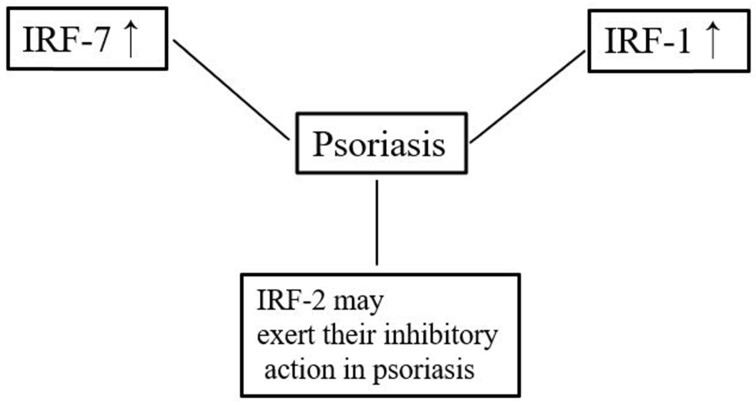
Overview of the relationships between TLR signaling and psoriasis.

**Figure 3:**
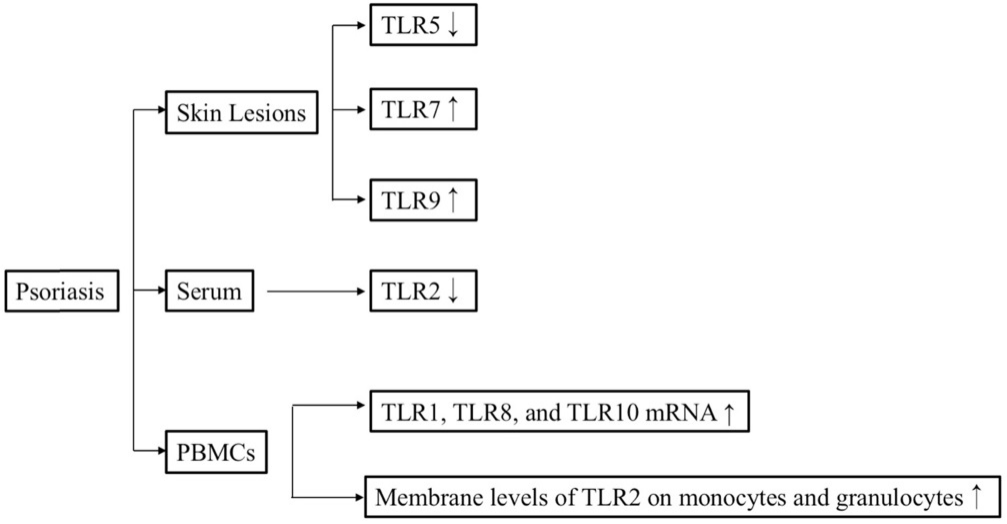
Overview of the relationships between IRFs and psoriasis.
